# ATP-competitive Plk1 inhibitors induce caspase 3-mediated Plk1 cleavage and activation in hematopoietic cell lines

**DOI:** 10.18632/oncotarget.23650

**Published:** 2017-12-23

**Authors:** Maeva Dufies, Damien Ambrosetti, Sonia Boulakirba, Anne Calleja, Coline Savy, Nathan Furstoss, Marwa Zerhouni, Julien Parola, Lazaro Aira-Diaz, Sandrine Marchetti, Francois Orange, Sandra Lacas-Gervais, Frederic Luciano, Arnaud Jacquel, Guillaume Robert, Gilles Pagès, Patrick Auberger

**Affiliations:** ^1^ Université Côte d’Azur, C3M/Inserm U1065, Nice, France; ^2^ Université Côte d’Azur, Institute for Research on Cancer and Aging of Nice (IRCAN), CNRS UMR 7284, INSERM U 1081, Nice, France; ^3^ Université Côte d’Azur, CHU Nice, Department of Pathology, Nice, France; ^4^ Equipe Labellisée par la Fondation ARC (2017-2020), Paris, France; ^5^ Université Côte d’Azur, CCMA, Nice, France

**Keywords:** Plk1, caspases, hematopoietic cells, G2M arrest, mitotic catastrophe

## Abstract

Polo-like kinases (Plks) define a highly conserved family of Ser/Thr kinases with crucial roles in the regulation of cell division. Here we show that Plk1 is cleaved by caspase 3, but not by other caspases in different hematopoietic cell lines treated with competitive inhibitors of the ATP-binding pocket of Plk1. Intriguingly, Plk1 was not cleaved in cells treated with Rigosertib, a non-competitive inhibitor of Plk1, suggesting that binding of the inhibitor to the ATP binding pocket of Plk1 triggers a conformational change and unmasks a cryptic caspase 3 cleavage site on the protein. Cleavage occurs after Asp-404 in a DYSD/K sequence and separates the kinase domain from the two PBDs of Plk1. All Plk1 inhibitors triggered G2/M arrest, activation of caspases 2 and 3, polyploidy, multiple nuclei and mitotic catastrophe, albeit at higher concentrations in the case of Rigosertib. Upon BI-2536 treatment, Plk1 cleavage occurred only in the cytosolic fraction and cleaved Plk1 accumulated in this subcellular compartment. Importantly, the cleaved N-Terminal fragment of Plk1 exhibited a higher enzymatic activity than its non-cleaved counterpart and accumulated into the cytoplasm conversely to the full length and the C-Terminal Plk1 fragments that were found essentially into the nucleus. Finally, the DYSD/K cleavage site was highly conserved during evolution from *c. elegans* to human. In conclusion, we described herein for the first time a specific cleavage of Plk1 by caspase 3 following treatment of cancer cells with ATP-competitive inhibitors of Plk1.

## INTRODUCTION

Caspases are cysteine proteases that play an important role in the regulation of cellular homeostasis including cell death, differentiation and inflammation. They cleave numerous of proteins that play diverse roles in the execution and regulation of these different cellular processes [1]. Several hundred caspase substrates have been identified, the function of which in the above-mentioned processes are far from being completely understood [2].

Most of the time, caspase cleavage triggers inactivation of protein substrates responsible for cell death demise [3]. However, in some circumstances, cleavage occurs between distinctive constitutive domains of the protein substrate leading to modulation of its activity [4]. This is particularly true regarding transcription factors such as serum responsive factor, for which caspase 3-dependent cleavage separates the DNA binding domain from the transcription activation domain, thus modulating its function [5]. This is also exemplified by protein kinases, for which caspase 3 cleavage between different structural domains, may lead to deregulation of the enzymatic kinase activity. This type of cleavage has been noticeably reported for the Src tyrosine kinases Lyn and Fyn, in which cleavage after Asp-18 or Asp-19 respectively by caspase 3 eliminates the N-Terminal anchoring domain, thereby generating a more active form kinase that delocalizes into the cytoplasm [6–8].

Plks (Plk1 to 4) are essential serine/threonine kinases that play a plethora of functions in the regulation of cell division and proliferation [9]. Plk1 is a highly conserved protein and orthologues have been identified from budding yeast to humans [10]. All Plks are characterized by the presence of a tandem of 2 highly conserved regions, called the Polo-Box Domains (PBDs) [11]. These PBDs are required to target their kinase domains to their specific substrates [12]. Excepted Plk5, all Plks harbor a highly conserved N-Terminal serine/threonine kinase domain that is essential for their functions [13]. In mammals, Plk1 acts at different stages of the M phase of the cell cycle and exerts important role in centrosome, kinetochore and midbody regulation [14–17]. The PDBs are crucial for all these functions of Plk1, since their deletion inhibits the effect of the kinase on cell cycle and division [18]. The complex interaction between the kinase domain and the PDB domain of Plk1, and their common regulation allowing Plk1 to specifically activate its substrates has been well studied and characterized. Interestingly, it has been proposed that the PBDs in an inactive state inhibit the kinase activity of Plk1. Finally, Plk1 is also tightly regulated by post-translational mechanisms including phosphorylation and degradation by the proteasome [19].

Low expression of Plk1 is a feature of non-proliferating cells while increased expression of Plk1 is associated with highly proliferating cells and is often linked to oncogenesis of human cells [20, 21]. Thus, Plk1 inhibitors efficiently kill cancer cells *in vitro* but have no effect in untransformed cells. Accordingly, high expression of Plk1 is associated with higher aggressiveness in different types of cancers [22, 23].

In the present study, we described for the first time that Plk1 is specifically cleaved by caspase 3 but not by other caspases in different human hematopoietic cell lines. Importantly, the cleavage separates the kinase domain from the PDBs and generates a 44 kDa N-Terminal cytosolic kinase harboring an increased enzymatic activity of Plk1. This fragment is retained into the cytosol while the remaining C-Terminal 25 kDa fragment localizes into the nucleus.

## RESULTS

### ATP-directed inhibitors of Plk1 triggers Plk1 cleavage

**Figure 1:**
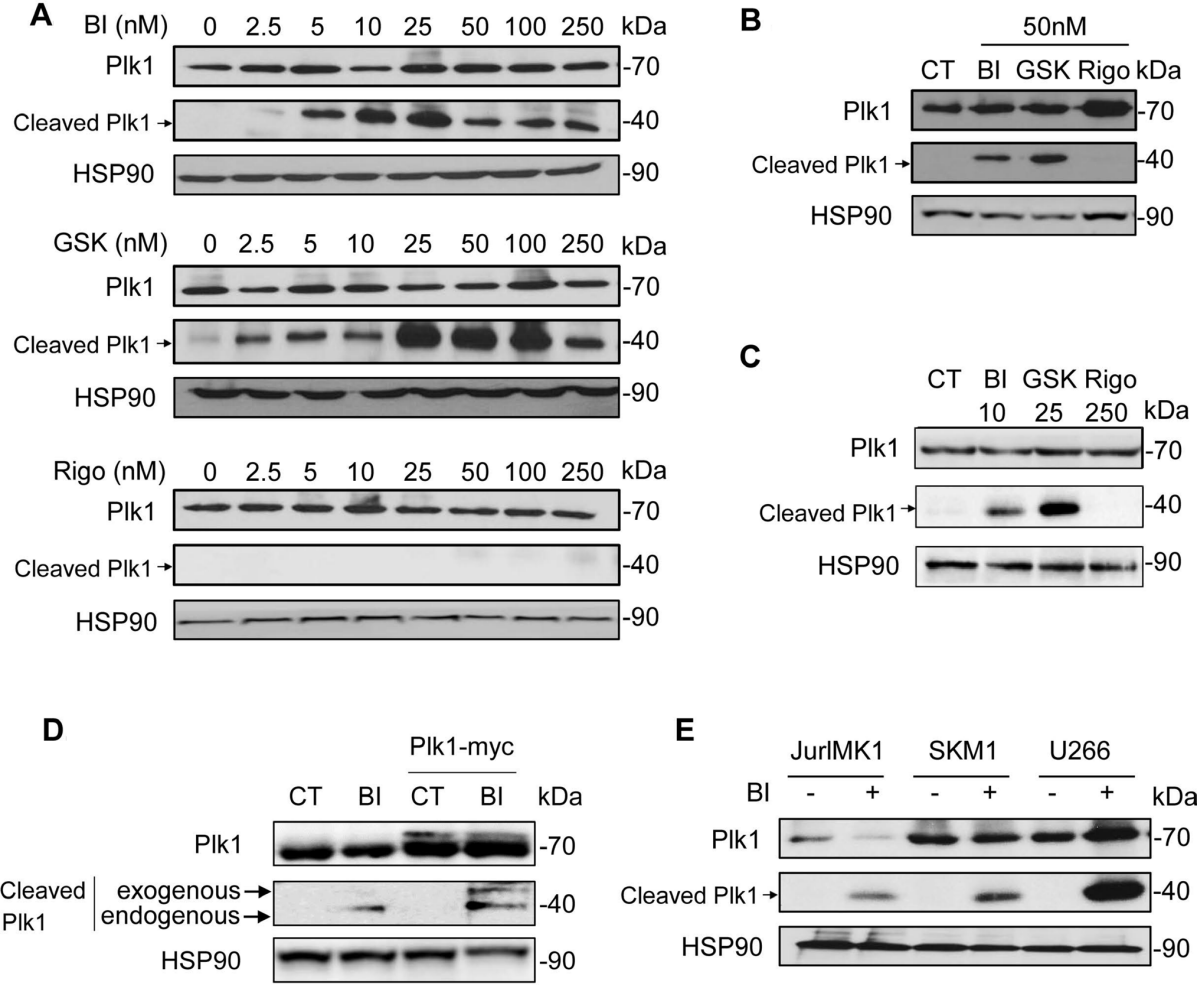
ATP-directed but not allosteric inhibitors of Plk1 triggers Plk1 cleavage (**A–B**) K562 cells were incubated for 48 h with increasing concentrations of BI-2536 (BI), GSK-431363 (GSK), and Rigosertib (Rigo) (A), or with 50 nM of BI-2536, GSK-431363, and Rigosertib (B), or with 10 nM BI-2536, 25 nM GSK-431363 and 250 nM Rigosertib (**C**). Expression and cleavage of Plk1 were analysed by Western blot. (**D**) Plk1-myc plasmid was transfected in K562. After 48 h, cells were treated with 25 nM BI-2536 for 48 h. Expression and cleavage of Plk1 were analysed by Western blot (Plk1 antibody). (**E**) JURLMK1 another CML cell line, SKM1 an MDS/AML cell line and the multiple myeloma U266 cell line were treated with 25 nM BI-2536 for 48 h. Expression and cleavage of Plk1 were analysed by Western blot. Each panel is representative of at least 3 independent experiments.

We investigated the effect of three Plk1 inhibitors (BI-2536, GSK-461363 and Rigosertib) on Plk1 expression in the chronic myelogenous cell line (CML) K562. Interestingly, BI-2536 and GSK-461363, two ATP-competitive inhibitors of Plk1 induced a dose-dependent cleavage of Plk1, generating a characteristic 44 kDa fragment (Figure 1A). Intriguingly and conversely to BI-2536 and GSK-461363, Rigosertib (ON-01910), an allosteric inhibitor of Plk1 failed to trigger Plk1 cleavage even at 10 to 50-fold higher doses as compared to BI-2536 (Figure 1A). The absence of Plk1 cleavage by Rigosertib was further confirmed on K562 cells treated with an equivalent dose of the three inhibitors (50 nM) (Figure 1B). Moreover, an equivalent cleavage was detected following BI-2536 or GSK-461363 treatment at 10nM and 25 nM respectively, while Rigosertib at 250 nM had no effect (Figure 1C), confirming, in different experimental conditions the results presented in Figure 1A and 1B.

We next transfected K562 cells with a myc-tagged-Plk1 plasmid and we analyzed Plk1 cleavage following BI-2536 treatment. As expected, Plk1 was cleaved in untransfected K562 cells generating the characteristic 44 kDa band. In Plk1-transfected cells, BI-2536 triggered the cleavage of both exogenous and endogenous forms of Plk1 generating two bands with different molecular weight (47 and 44 kDa), corresponding to exogenous Myc-tagged Plk1 and endogenous Plk1 fragments, respectively (Figure 1D). Finally, we detected the cleavage of Plk1 in three other cell lines, JURLMK1, another CML cell line, SKM1 an MDS/AML (myelodysplastic syndrome/acute myeloid leukemia) cell line and U266, a multiple myeloma cell line (Figure 1E). This result confirms that the cleavage of Plk1 induced by ATP-competitive inhibitors of Plk1 may occur in different hematopoietic cell lines. This result was further confirmed in a subset of non-hematopoietic cell lines, including RCC4, a clear cell renal cell carcinoma cell line (Supplementary Figure 1).

### Caspase 3 is responsible for Plk1 cleavage

**Figure 2:**
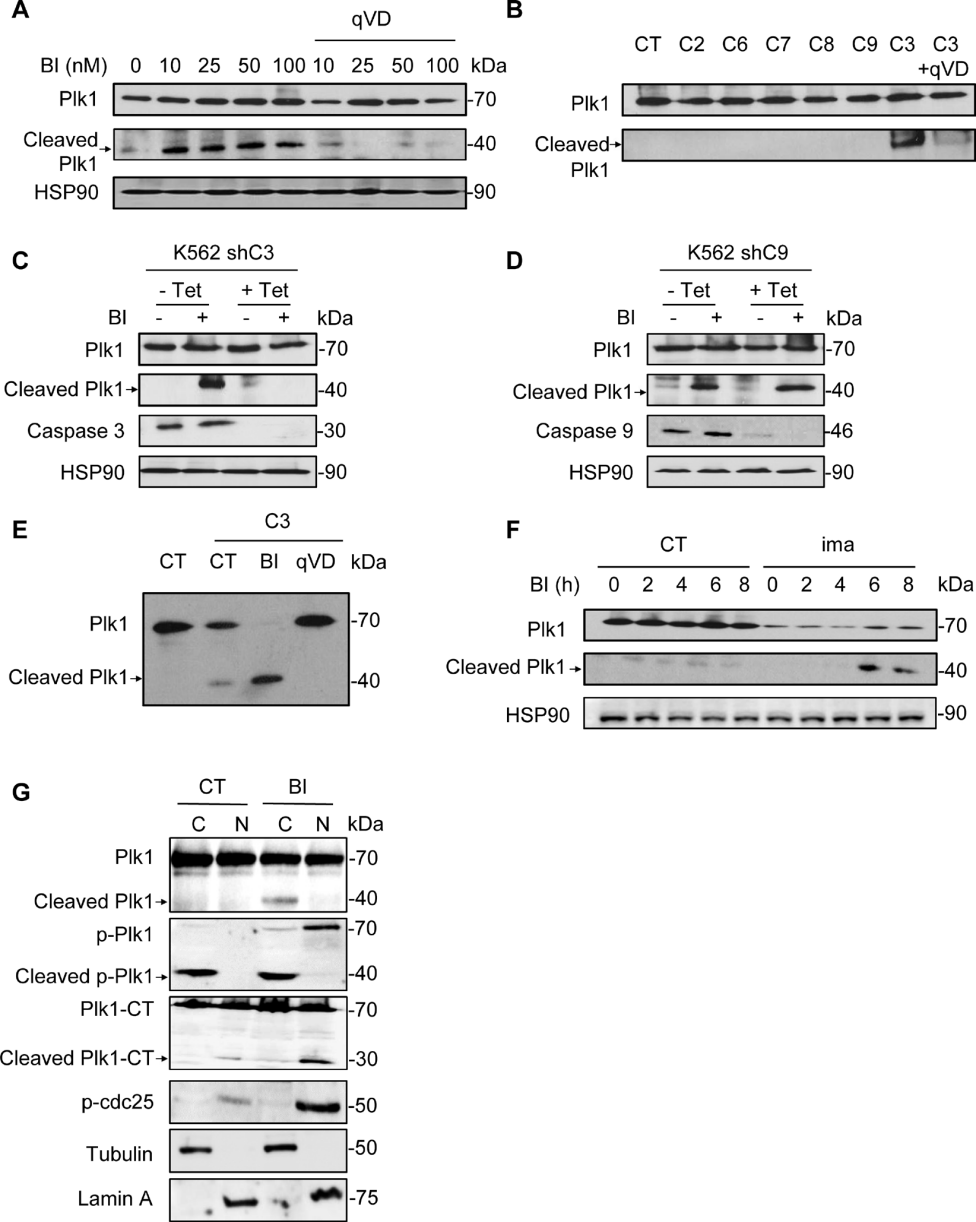
Caspase 3 is responsible for Plk1 cleavage (**A**) K562 cells were incubated for 48 h with increasing concentrations of BI-2536 in presence of 50 µM qVD. Expression and cleavage of Plk1 were analysed by Western blot. (**B**) Plk1 was transcribed/translated in a TNT assay and the generated Plk1 protein is incubated with indicated recombinant caspases (C2, C3 and C6 to C9) in the presence or absence of 50 μM qVD. Expression and cleavage of Plk1 were analyzed by Western blot. (**C** and **D**) After a 5 day pre-treatment with 1 μg/ml tetracycline to allow knock-down of caspase 3 (K562 shC3, C) or 9 (K562 shC9, D), K562 cells were cultured for 48h with or without 25 nM BI-2536. Expression and cleavage of Plk1, and expression of caspase 3 and caspase 9 were analysed by Western blot. (**E**) Plk1 was transcribed/translated in a TNT assay and the generated Plk1 protein is incubated with recombinant caspase 3 in the presence or absence of 50 μM qVD or 50 nM BI-2536. Expression and cleavage of Plk1 were analysed by Western blot. (**F**) K562 cells were pre-treated with 1 µM Imatinib (ima) for 24 h. Then, cells were treated with 25 nM BI-2536 for 0 to 8 h. Expression and cleavage of Plk1 were analyzed by Western blot. (**G**) Cells were treated with 25 nM BI-2536 for 24 h. Next, cell extracts were separated into cytosol and nuclear-enriched fractions and Plk1 location (Plk1, p-Plk1 and Plk1-CT antibody) was assessed by Western blot in the different fractions. Lamin A and Tubulin served as both loading control and validation of the nuclear and cytoplasmic fractions, respectively. p-Plk1 antibody recognizes the activated (phospho-threonine 210) N-Terminal (NT) epitope and cleavage product NT, and Plk-CT recognizes the C-Terminal epitope and corresponding cleavage product CT. Each panel is representative of at least 3 independent experiments.

Plk1 inhibitors have been previously shown to induce apoptosis in different hematopoietic and non-hematopoietic cell lines. We therefore hypothesized that Plk1 cleavage could be the result of caspase activation. To investigate this possibility, K562 cells were pretreated with the pan-caspase inhibitor qVD prior to BI-2536 addition for 24 h. qVD abolished Plk1 cleavage, whatever the concentration of BI-2536 used, suggesting that caspases are involved in Plk1 processing (Figure 2A and Supplementary Figure 2C). Induction of apoptosis by Plk1 inhibitors was further demonstrated by an increase in PARP cleavage in two different hematopoietic cell lines K562 and U266 (Supplementary Figure 2A, 2B and 2D). We next investigated whether Plk1 could be cleaved by individual recombinant caspases. To this aim, Plk1 was transcribed/translated in a TNT assay and the generated Plk1 protein was next subjected to cleavage by either purified caspase 2, 3, 6, 7, 8 or 9. Among the six apoptotic caspases, only caspase 3 cleaved Plk1 in this *in vitro* assay and the cleavage was impaired in the presence of qVD (Figure 2B).

To further characterize the cleavage of Plk1 we used K562 cells in which expression of caspase 3 or caspase 9 is down-regulated by specific shRNA upon doxycycline treatment. This cell lines have been previously described [24]. After three days of doxycycline treatment, caspase 3 or caspase 9 proteins were almost undetectable (Figure 2C and 2D). Interestingly, cleavage of Plk1 was abolished in K562 cells invalidated for caspase 3 (Figure 2C) but not for caspase 9 (Figure 2D), confirming the crucial role of caspase 3 in this cleavage of Plk1 and the results of our *in vitro* experiments with recombinant caspase 9 (Figure 2B). We further assessed the effect of BI-2536 in the cell free system described above and interestingly, we found that addition of BI-2536 increased strikingly Plk1 cleavage by caspase 3 (Figure 2E). Identical results were obtained in K562 and U266 cells using Ac-DEVD-CHO, a specific inhibitor of caspase 3, further confirming the involvement of caspase 3 in Plk1 cleavage (Supplementary Figure 3A and 3B). We also checked that Ac-DEVD-CHO was as efficient as qVD to impair caspase 3 activity (Supplementary Figure 3C). In addition, Plk1 cleavage was confirmed in K562 cells pretreated with Imatinib (ima), a tyrosine kinase inhibitor, known to activate caspase 3 in these cells through inhibition of the tyrosine kinase activity of the Bcr-Abl fusion protein [25]. After 16 h of Imatinib treatment, a time sufficient to induce caspase 3 activation but not apoptosis in K562 cells, cells were treated at different times with 10 nM BI-2536. Pre-activation of caspase 3 by Imatinib triggered a very rapid cleavage of Plk1, which was detected after only 6h of incubation with BI-2536 versus 24–48 h in non-apoptosis primed cells (Figure 2F). We also verified in this experiment that Plk1 cleavage was abrogated in the presence of Ac-DEVD-CHO (Supplementary Figure 3D). These findings are compatible with a modification of the kinase conformation that unmasks a cryptic caspase 3 cleavage site. This could also explain why an allosteric Plk1 inhibitor such as Rigosertib fails to do so.

To gain insights into the subcellular localization of Plk1 following caspase 3 cleavage, protein extracts from control and BI-2536-treated K562 cells were separated in cytosolic and nuclear fractions. The purity of each fractions was assessed using adequate protein markers i.e Tubulin and Lamin A (Figure 2G). In untreated cells, Plk1 was present among the two fractions. BI-2536 induced Plk1 cleavage at 24 h and the 44 kDa Plk1 cleaved form accumulated solely in the cytoplasmic fraction. The 25 kDa C-Terminal fragment of Plk1 was found only in the nuclear fraction and accumulated more in BI treated cells. A phosphorylated cleaved form of Plk1 accumulated in the cytoplasmic fraction in BI-2536-treated conditions (Figure 2G). Intriguingly, cleavage could also occur at a lower rate in untreated cells. In BI-2536-treated cells, the full-length phosphorylated form of Plk1 increased in both fractions albeit to a higher level than in the nuclear fraction (Figure 2G). These results were further confirmed in U266 cells (Supplementary Figure 4). Of note, using an antibody directed against the C-Terminal domain of Plk1, we identified a 25 kDa band corresponding to the C-Terminal fragment of Plk1 (Supplementary Figure 4). For an unknown reason, BI-2536 treatment also increased phosphorylation of CDC25, a direct substrate of Plk1 in the nuclear fraction (Figure 2G and Supplementary Figure 4).

### The caspase 3 cleavage site in Plk1 is highly conserved during evolution

**Figure 3:**
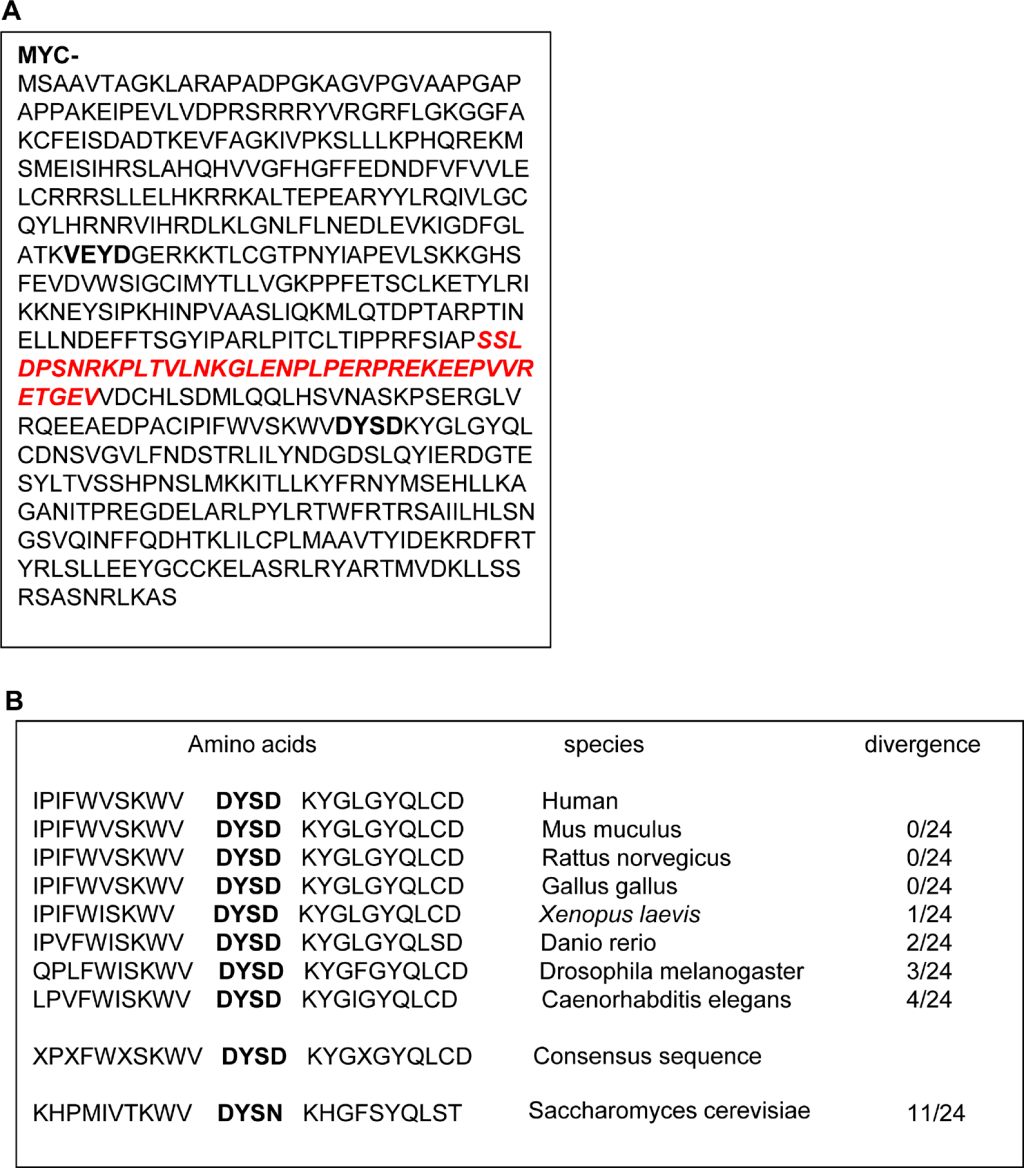
Plk1 cleavage sequence is highly conserved in phylogeny (**A**) Protein sequence of Plk1. caspase cleavage sites are indicated in bold. (**B**) The sequence alignment was performed using the protein sequence of different species given by PUBMED: http://www.ncbi.nlm.nih.gov/PubMed. The site recognized by the antibody is indicated in italic.

Figure 3A shows the complete amino acid sequence of the human Plk1 protein. Plk1 mRNA encodes a 603 amino-acid protein with a molecular weight of 66 kDa. Scrupulous examination of the amino acid sequence of Plk1 indicates that two aspartates located in position 204 and 404 have the prerequisite to be cleaved by caspases. Indeed, VEYD_204_/G and DYSD_404_/K in Plk1 behave as consensus cleavage sites for caspase 6 and caspase 3, respectively. However, the data illustrated in Figure 2B and 2C, and in Supplementary Figure 3 clearly indicate that only caspase 3 cleaves Plk1. We therefore concluded that Plk1 is cleaved after Asp404 by caspase 3 in different cell lines treated with ATP-competitive Plk1 inhibitors, in agreement with the detected sizes of the Plk1 fragments at 44 and 25 kDa. Importantly, the consensus site for caspase 3 was also highly conserved along evolution from *Caenorhabditis elegans* to human with only very minor variations in the amino acids surrounding Asp-404 (Figure 3B). This finding suggests that this cleavage site may have a physiological relevance.

### Plk1 inhibitors induced loss of cell viability, increased caspase 3 activity, accumulation in the G2M phase of the cell cycle and apoptosis

**Figure 4:**
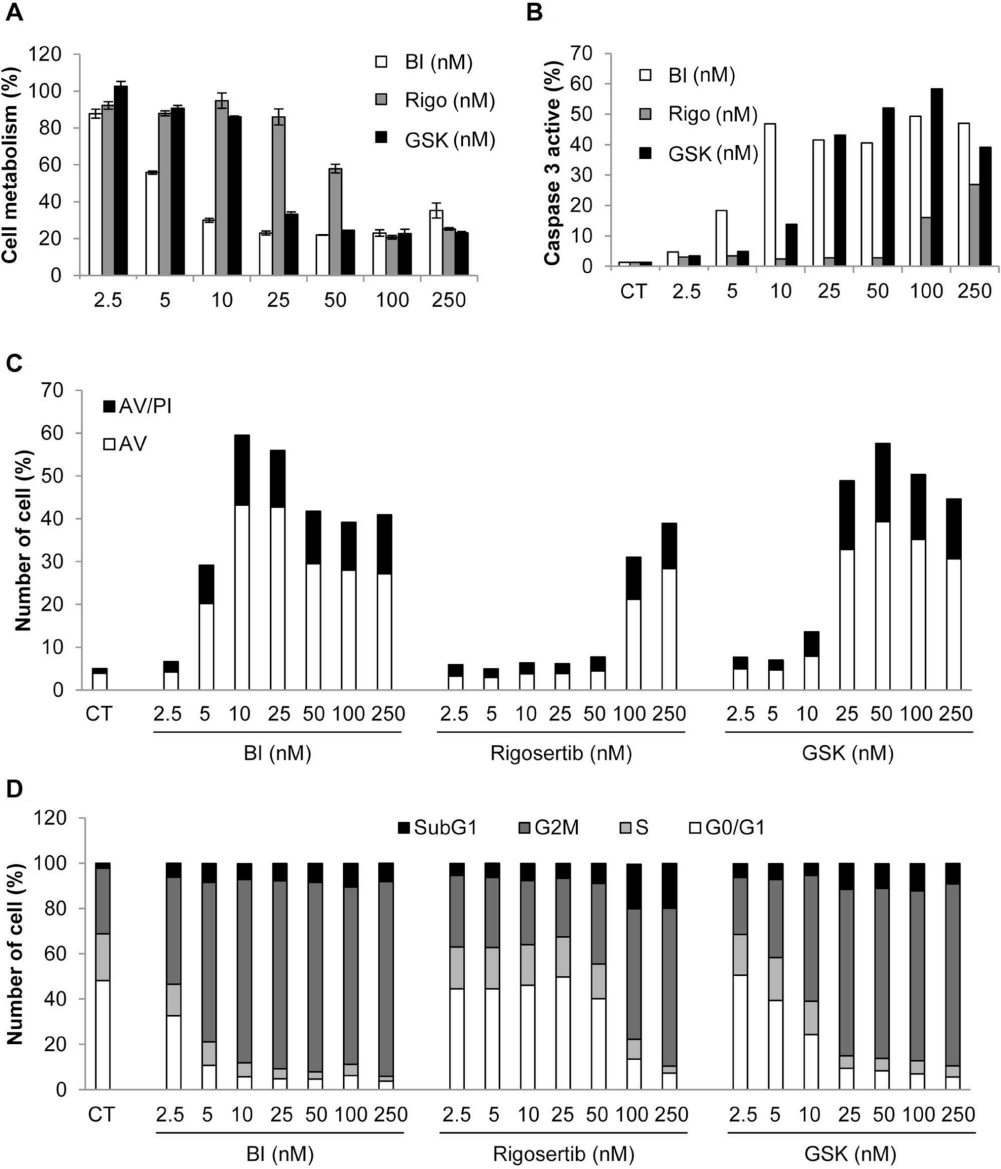
Plk1 inhibitors induce loss of cell viability, increased caspase 3 activity, apoptosis, and accumulation in the G2M phase of the cell cycle K562 cells were incubated for 48 h with increasing concentrations of BI-2536, GSK-431363, and Rigosertib. (**A**) Cell metabolism was measured using the XTT assay. (**B**) Cells were stained with an active caspase 3 antibody and analyzed by flow cytometry. (**C**) Cells were stained with the PI/ annexin-V-fluos staining kit. Histograms show both annexin-V+/PI- cells (open bars) and annexin-V+/PI+ cells (filled bars). (**D**) Cells were labelled for 15 min with PI and immediately analysed by flow cytometry. Histograms represent the percentage of cells in each phase of the cell cycle (subG1, G0/G1, S and G2/M). Each panel is representative of at least 3 independent experiments.

**Figure 5:**
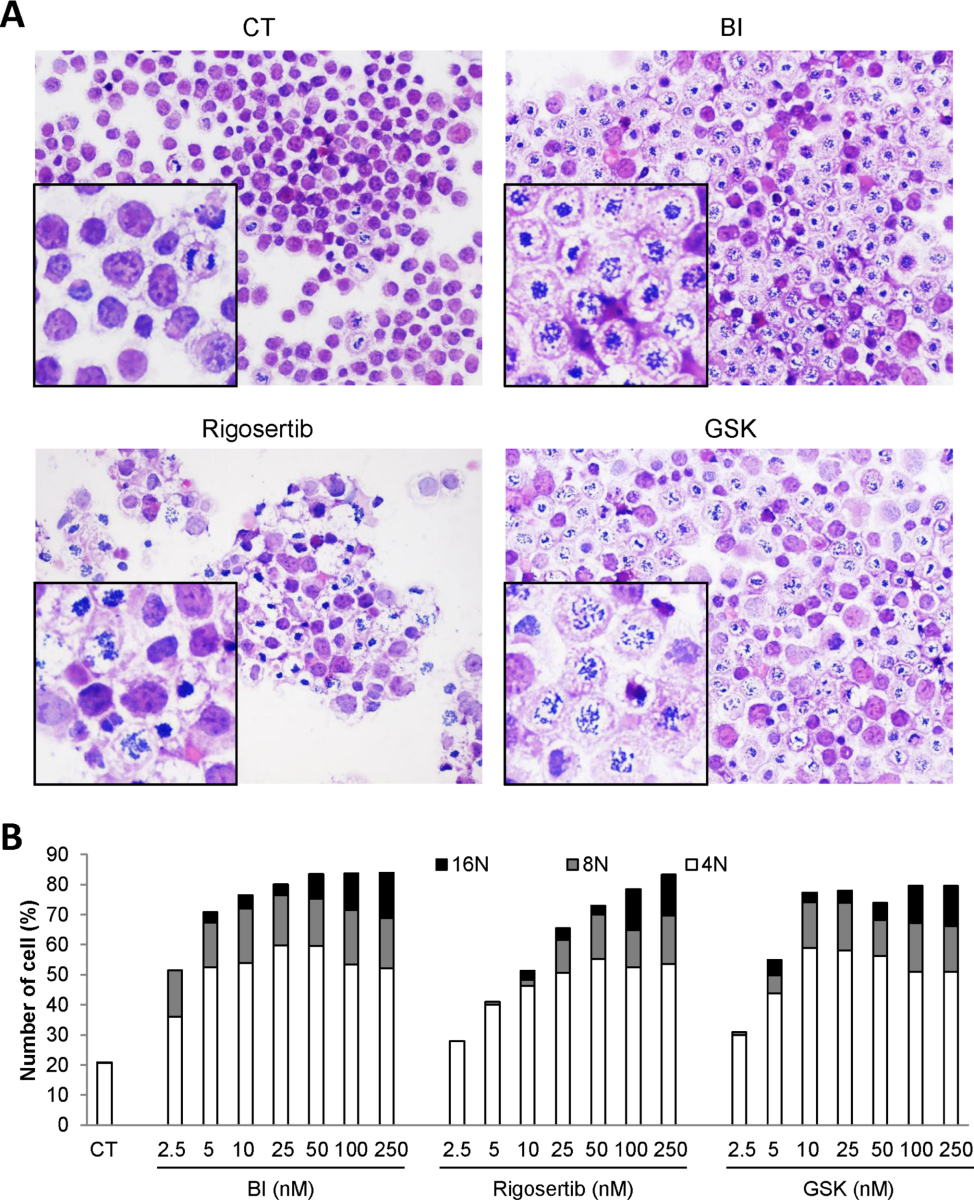
Plk1 inhibitors induce mitotic catastrophe (**A** and **B**) K562 cells were incubated for 24 h with 25 nM of BI-2536, GSK-431363, and Rigosertib. (A) Hematoxilin and Eosin (HE) staining was assessed. (B) Cells were labelled for 15 min with PI and immediately analysed by flow cytometry. Histograms represent the percentage of cells with DNA content of 4N, 8N and 16N. Each panel is representative of at least 3 independent experiments.

Plk1 inhibitors have been previously reported to induce loss of cell viability and apoptosis in different cancer cell lines [26]. We next analyzed the effects of Plk1 inhibitors on the viability of the K562 cell line. All Plk1 inhibitors induced a dose-dependent inhibition of K562 cell viability. Maximal inhibition (80%) was obtained with 10 nM BI-2536, 50 nM GSK-461363 and 100 nM Rigosertib, respectively. The IC50 values for BI-2536, GSK-461363 and Rigosertib were 6, 20, and 55 nM, respectively (Figure 4A). Loss of cell viability was strictly correlated to an increase in caspase 3 activity induced by the three Plk1 inhibitors as assessed by flow cytometry (Figure 4B). The IC50 values for caspase 3 activation were 5, 25 and 250 nM for BI-2536, GSK-461363 and Rigosertib, respectively. Most of the Plk1 inhibitor effect on cell viability was accounted for by apoptosis induction as shown by an increased proportion of cell stained with Annexin V (Figure 4C). Globally, increased caspase 3 activation and Annexin V staining further indicate a major role of apoptosis in the anti-leukemic effect of Plk1 inhibitors. It has been previously reported that Plk1 inhibition leads to cell cycle arrest in the G2 phase of the cell cycle [27]. This was confirmed in K562 cells where low doses of BI-2536, GSK-461363 and Rigosertib triggered G2/M arrest at the same concentrations than caspase 3 activation and apoptosis induction (Figure 4D). Finally, inhibition of Plk1 by the three inhibitors induces a major defect in cell division resulting in several aberrant mitosis according to the cytology analyses (Figure 5A). Plk1 inhibition also increased the nuclear DNA content of K562 cells to 8N and 16N (Figure 5B) which is reminiscent of mitotic catastrophe. Induction of mitotic catastrophe was further confirmed using transmission electron microscopy. Indeed, electron microscopy images demonstrated that BI-2536-treated cells exhibited multiple nuclei characteristic of mitotic catastrophe induction compared to untreated cells (Supplementary Figure 5). Caspase 2 has been recently reported to be induced during mitotic catastrophe [28–30]. Accordingly, we found that caspase 2 was activated before caspase 3 in K562 cells treated with Plk1 inhibitors, confirming the possible involvement of this caspase in the induction of mitotic catastrophe by these molecules (Supplementary Figure 6).

### Volasertib activates caspase 2 and 3 and induces polyploidy in K562 cells

**Figure 6:**
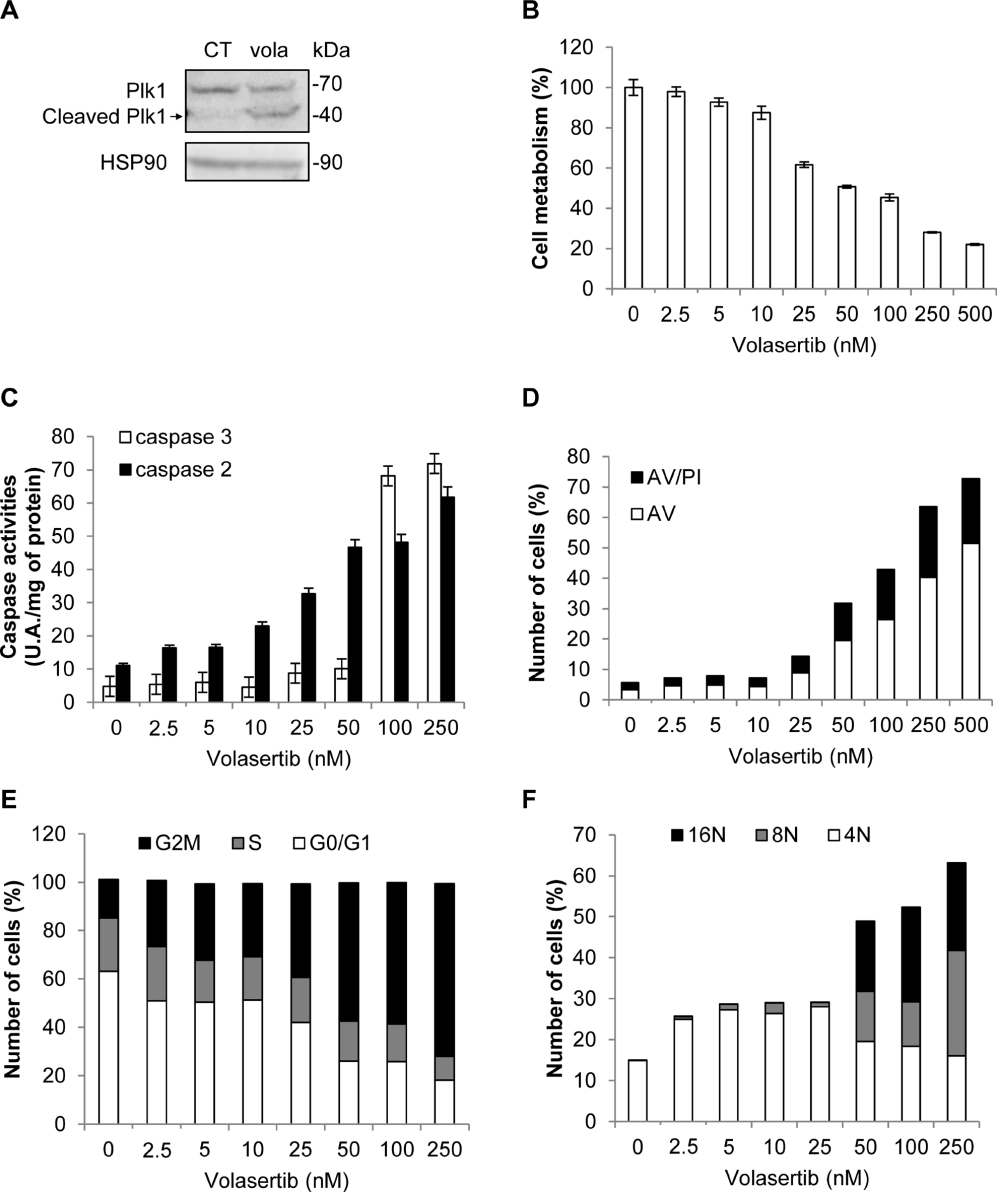
Volasertib activates caspase 2 and 3 and induces polyploidy in K562 cells (**A**) K562 cells were incubated for 48 h with 50 nM of Volasertib. Expression and cleavage of Plk1 were analyzed by Western blot. (**B–F**) K562 cells were incubated for 48 h with increasing concentrations of Volasertib. (B) Cell metabolism was measured using the XTT assay. (C) Cells were lysed in caspase buffer and caspase-3 and -2 activities were evaluated in quadruplicate using 0.2 mM Ac-DEVD-AMC or Ac-VDVAD-AMC respectively as substrates. Results expressed as arbitrary units (A.U.)/min per mg of protein. (D) Cells were stained with the PI/Annexin-V-fluos staining kit. Histograms show both Annexin-V+/PI- cells (open bars) and Annexin-V+/PI+ cells (filled bars). (E and F) Cells were labelled for 15 min with PI and immediately analysed by flow cytometry. Histograms represent the percentage of cells in each phase of the cell cycle (G0/G1, S and G2/M) (**E**), or cells with DNA content of 4N, 8N and 16N (F). Each panel is representative of at least 3 independent experiments.

Volasertib (BI-6727) is an ATP-competitive Plk1 inhibitor which has entered phase III clinical trials in AML in combination with cytarabine [31]. Like BI-2536, Volasertib induces the cleavage of Plk1 (Figure 6A). Volasertib dose-dependently decreased K562 viability with a maximal effect at 500 nM. The IC50 value for Volasertib in K562 was 50 nM (Figure 6B). It also triggered apoptosis in K562 cells as shown by a rise in caspase 3 activity in the 50 to 250 nM range (Figure 6C) and a concomitant increase in Annexin V staining (Figure 6D). Beside caspase 3, Volasertib increased caspase 2 activity at concentrations for which caspase 3 was not yet active (Figure 6C). In addition, Volasertib also promoted G2/M arrest of K562 cells, which is consistent with the effects of the other Plk1 inhibitors (Figure 6E). Furthermore, Volasertib triggered polyploidization of K562 cells as attested by an increase in the proportion of cells with 8N and 16N DNA content at 24 h (Figure 6F). Finally, the cleavage of Plk1 with BI-2536 and Volasertib, but not Rigosertib was confirmed in primary myeloid cells from one CML patient (Supplementary Figure 7).

## DISCUSSION

The Polo-like kinases define a highly conserved family of Ser/Thr kinases that play essential roles in the regulation of the cell cycle. They include three closely-related proteins Plk1, 2 and 3 and a more distant member Plk4 [32]. Besides the presence of the 44 kDa N-Terminal kinase domain essential for the function of the protein, one of the characteristics of Plks is the presence of two Polo-Box Domains (PBD1 and PDB2) in the 25 kDa C-Terminal part of the protein. Plk1 plays essential roles at multiple stages of the M phase, including centrosome maturation, mitotic entry and cytokinesis. Plk1 is also a very attractive target for cancer therapy because it is overexpressed in most tumor cells and tissues and poorly or not expressed in untransformed cells [33].

In the present study, we show for the first time that Plk1 is cleaved by caspase 3 upon treatment with Plk1 ATP-competitive inhibitors but not upon treatment with a non-competitive allosteric inhibitor of Plk1, Rigosertib, in different leukemic cell lines. Rigosertib is a small molecule inhibitor that inhibits the RAS, PI3K and Plk1 signaling pathways [34]. It exerts potent antineoplastic activity in multiple tumor cell lines and is currently tested in phase III clinical trial for chronic myelomonocytic leukemia [35]. Conversely to ATP-competitive inhibitors, Rigosertib inhibits Plk1 by competing at the substrate binding site. Recently, it was reported to disrupt the interaction of Plk1 with Ras leading to its anti-tumoral effects in tumor cells. From these findings, we conclude that direct interaction of the ATP-competitive Plk1 inhibitor with Plk1 is a prerequisite for Plk1 cleavage following caspase 3 activation. The non-competitive inhibitor of Plk1, Rigosertib, fails to induce Plk1 cleavage. It is possible that it does not trigger the conformational change necessary to expose the cryptic caspase 3 site located in the full-length protein. Interestingly following the cleavage of Plk1, the fate of the two cleaved fragments are completely different. The N-Terminal kinase domain accumulated in the cytosol in an active conformation, while the C-Terminal fragment containing the two PDB domains is distributed within the nucleus. It would be very interesting in future studies to analyze precisely the function of the two fragments of Plk1 in their respective localization and how this can impact Plk1 function.

An interesting mechanism that could arise from the model presented in our study is that it could exist a cell intrinsic mechanism of Plk1 cleavage when the kinase encounters its specific substrates. In such a model, binding of the substrates to the PDB domains of Plk1 would trigger exposition of the cryptic caspase 3, ultimately conferring caspase 3 sensitivity. Of note, Plk1 is not cleaved following treatment with other pro-apoptotic agents including staurosporine or Imatinib in K562 cells or Velcade in the U266 multiple myeloma cell line. However, it is rapidly cleaved following treatment with by ATP-competitive inhibitors of Plk1 in cells primed for apoptosis i.e. K562 cells pretreated with Imatinib (Figure 2F). This finding suggests that the caspase 3 site is masked in endogenous Plk1 in the absence of inhibitors.

Of note, Plk1 is specifically cleaved by caspase 3, but not other caspases, thus generating N-Terminal 44 kDa and C-Terminal 25 kDa fragments (Supplementary Figure 4D). Cleavage occurs after Asp-404. The longest (aa 1-404) comprises the kinase domain and the shortest (aa 405-603) the two PBD domains, thus resembling the inactive kinase Plk5. Conversely to other Plks, Plk5 has no established role in cell cycle regulation and some data indicate that it could act as a tumor suppressor [36, 37], since its expression diminishes in several cancer cell lines. In addition, it has been reported that overexpression of Plk5 in neurons induces apoptosis [36]. There is compelling evidences that the PDB of Plks and more particularly PDB1 of Plk1 regulates the function of its own kinase activity through interaction between the PDB1 domain and the kinase domain [38]. These findings suggest that the PDB1 binding domain and the kinase domain interact with each other in a mutually inhibitory fashion. Here we showed that Plk1 inhibitors triggered the cleavage of Plk1 at aspartate 404 and separated the kinase domain from the PDB domains of Plk1. This cleavage leads to the generation of a kinase which localizes essentially into the cytoplasm. This cleaved form was found to be highly auto-phosphorylated, suggesting that it corresponds to an active kinase. This finding is consistent with the above-mentioned inhibitory effect of the PDBs on Plk1 kinase activity and consistent with the fact that elimination of the PDBs domains following caspase 3 cleavage, leads to a constitutive activation of Plk1.

It is also particularly interesting that Plk1 is not cleaved when caspase 3 is activated by different pro-apoptotic stimuli that does not directly affect Plk1 or by non-competitive inhibitors of Plk1 such as Rigosertib, whereas cleavage is induced by ATP-competitive Plk1 inhibitor. This result strongly suggests that binding of ATP-competitive inhibitors to the ATP binding pocket of Plk1 induces a conformational change in the kinase domain that unmasks a caspase 3 cleavage site on the protein allowing cleavage at aspartate 404. This finding is also supported by the fact that caspase 3 pre-activation in K562 cells by Imatinib for instance accelerates Plk1 cleavage in the presence of BI-2536.

Finally, we confirmed in the present study that another ATP-competitive inhibitor of Plk1, Volasertib, promoted G2M arrest, mitotic catastrophe, caspase 2 and 3 activation and finally Plk1 cleavage after aspartate 404. Volasertib is a small molecule inhibitor of Plk1 that has entered phase III clinical trial for AML in combination with cytarabine (clinicaltrials.gov, NCT01721876). It would be interesting in future experiments to analyze whether cleavage of Plk1 by caspase 3 upon Volasertib treatment is important for the effect of this drug.

We described here that all Plk1 inhibitors tested induced mitotic catastrophe, activation of caspase 2 and apoptosis in different hematopoietic cell lines. Caspase 2 next activated caspase 3 that ultimately cleaved Plk1. These findings are reminiscent of our recent results showing that foretinib, a multi-kinase inhibitor targeting Met, Ron, Axl and the VEGF receptor, triggered mitotic catastrophe, activation of caspase 2 and apoptosis in different hematopoietic cell lines, including K562 cells. In addition, we established that foretinib induces caspase 2-dependent apoptosis of K562 cells. Moreover, we found that foretinib inhibited the JNK pathway leading to proteosomal degradation of Plk1 independently of caspase cleavage, since cleavage persisted in cells treated with caspase inhibitors [28].

In conclusion, we described here a novel mechanism of Plk1 regulation that involves its specific cleavage by caspase 3 in hematopoietic cell lines. This cleavage is selectively triggered when ATP competitive inhibitors bind to the active site of the kinase. The cleavage separates the kinase domain from the PDB domain and results in different localization of the cleaved Plk1 domains in hematopoietic cells. The exact role of each Plk1 fragments upon caspase 3 cleavage would warrant further examination.

## MATERIALS AND METHODS

### Reagents and antibodies

Imatinib mesylate was purchased from Enzo Life Sciences. BI-2536, Rigosertib, GSK-461363 and volasetib were purchased from Selleckchem. RPMI 1640 medium, and fetal calf serum (FCS) were from life technologies. Sodium fluoride, sodium orthovanadate, phenyl-methyl-sulfonyl fluoride (PMSF), aprotinin, leupeptin were purchased Sigma- Aldrich. Anti-HSP60 and anti-HSP90 were purchased from Santa Cruz Biotechnology. HRP conjugated anti-mouse and anti-goat antibodies were from Dakopatts. Anti-PARP, anti-phospho-Plk1, anti-caspase 3, anti-caspase 9, anti-Tubulin, anti-phospho-CDC25 and peroxydase-conjugated anti-rabbit antibodies were obtained from Cell Signaling Technology. Anti-Plk1 was purchased from Abcam.

### Cell lines

Human CML cell lines K562 and JURLMK-1, MDS/AML cell line SKM1 and multiple myeloma cell line U266 were grown at 37°C under 5% CO 2 in RPMI supplemented with 10% FCS, 50 U/ml penicillin, 50 μg/ml streptomycin, and 1mM sodium pyruvate. K562-shC3 and K562-sh C9 cells have been previously described [24].

### Measurement of cell viability

Cells (15 × 10^3^ cells/100µl) were incubated in a 96 well-plate with different effectors for the times indicated in the figure legends. Fifty microliters of sodium 3-[1-phenylaminocarbonyl)-3,4-tetrazolium]- bis(4-methoxy-6-nitro) benzene sulfonic acid hydrate (XTT) reagent was added to each well. The assay is based on the cleavage of the yellow tetrazolium salt XTT to form an orange formazan dye by metabolically active cells. The absorbance of the formazan product, reflecting cell viability, was measured at 490 nm. Each assay was performed in quadruplicate.

### Caspase activities measurements

Caspase assays have been described elsewhere [28]. Briefly, following stimulation, cells were lysed for 30 min at 4°C in lysis buffer (50 mM HEPES pH 8, 150 mM NaCl, 20 mM EDTA, 1 mM PMSF, 10 µg/mL leupeptin, 10 µg/mL aprotinin and 0.2% Triton X-100) and lysates were cleared at 10 000g for 15 min at 4°C. Each assay (in triplicate) was performed with 50 µg of protein prepared from control or stimulated cells. Briefly, cellular extracts were then incubated in a 96-wells plate with 0.2 mM of DEVD-AMC (Caspase-3) or VDVAD-AMC (Caspase-2) as substrates for various times at 37°C. Caspase activity was measured following emission at 460 nm (excitation at 390 nm) in the presence or not of 10 µM of DEVD-CHO or VDVAD-CHO. Enzyme activities were expressed in arbitrary units (A.U.) per min and per mg of proteins.

### Flow cytometry analysis

#### Apoptosis assay

After stimulation, cells were washed with ice-cold PBS and were stained with the annexin-V-fluos staining kit (Roche, Meylan, 11858777001) according to the manufacturer procedure. Fluorescence was measured by using the FL2 channels of a fluorescence-activated cell sorter apparatus (Miltenyi cytometry).

#### Cell cycle assay

After treatment, cells were washed, fixed in ethanol 70% and, finally, left overnight at –20°C. Cells were next incubated in PBS, 3 μg/ml RNase A and 40 μg/ml of propidium iodide (PI) for 30 min at 4°C. Cell distribution across the different phases of the cell cycle or DNA content was analyzed with a MACSQuant® Analyzer (Miltenyi).

### Cytospin preparations

Cytospin preparations were carried out using the cytocentrifuge (Thermo Scientific Cytospin 4, Thermo, Pittsburgh, PA, USA) at 900 rpm for 9 minutes. Smears were stained with Hematoxylin–Eosin, for morphological assessment.

### Preparation of cytoplasmic and nuclear fractions

After stimulation, cells were washed and subcellular fractionation was performed as described previously in reference [39]. Proteins contained in both cytosolic and nuclear fractions were separated by SDS-PAGE and transferred onto PVDF membrane (Immobilon-P, Millipore, IPVH00010) before incubation with specific antibodies.

### *In vitro* transcription/translation of plk1 and cleavage by recombinant caspases

Plk1was transcribed and translated using the Promega TNT-coupled reticulocyte lysate system. Briefly, 25 ng recombinant caspases (Sigma) were incubated for 16 h at 37 °C with 2 μl of reticulocyte lysates in 50 μl of 25 mM HEPES buffer pH 7.5 containing 0.1% CHAPS and 5 mM dithiothreitol. In a subset of experiments, the caspase inhibitor qVD (50 μM) was preincubated with recombinant caspases before the addition of the reticulocyte lysates. Proteins were then electrophoresed on 12% PAGE and autoradiographed using Amersham Biosciences hyperfilms.

### Western blot analyzis

After stimulation, cells were harvested and lysed in buffer containing 1% Triton X-100 and supplemented with protease and phosphatase inhibitors (Roche Diagnostics). Lysates were pelleted, and 50μg of protein were analyzed by SDS-PAGE.

### Electron microscopy transmission

Electron Microscopy was performed on the CCMA EM Core Facility (Université Côte d’Azur). Cells were observed with transmission electron microscopy for ultrastructural characterisation. Cell pellets were fixed in a 1.6% glutataraldehyde solution in 0.1 M sodium phosphate buffer (pH 7.4) at room temperature and stored overnight at 4°C. After three rinsing in 0.1 M cacodylate buffer (15 min each), pellets were postfixed in a 1% osmium tetroxide and 1% potassium ferrocyanide solution in 0.1 M cacodylate buffer for 1 hour at room temperature. Cells were subsequently rinsed in DDW and dehydrated in a series of acetone baths (90%, 100% three times, 15 min each) and progressively embedded in Epon 812 (Fluka) resin (acetone / resin 1:1, 100% resin two times, 2 hours for each bath). Resin blocs were finally left to harden at 60°C in an oven for 2 days. Ultrathin sections (70 nm) were obtained with a Reichert Ultracut S ultramicrotome equipped with a Drukker International diamond knife and collected on 150 mesh copper grids. Sections were stained with lead citrate and uranyl acetate. TEM observations were performed with a JEOL JEM-1400 transmission electron microscope, equipped with a Morada camera, at a 100 kV acceleration voltage.

## SUPPLEMENTARY MATERIALS FIGURES


