# Supplementary material for: ATP-competitive Plk1 inhibitors induce caspase 3-mediated Plk1 cleavage and activation in hematopoietic cell lines
Source: Oncotarget. 2017 Dec 23;9(13):10920–33. doi: 10.18632/oncotarget.23650 (PMC5834281; doi:10.18632/oncotarget.23650)
Supplement: Supplementary file 1 [file oncotarget-09-10920-s001.pdf]

ATP-competitive Plk1 inhibitors induce caspase 3-mediated Plk1 cleavage and activation in hematopoietic cell lines

SUPPLEMENTARY MATERIALS

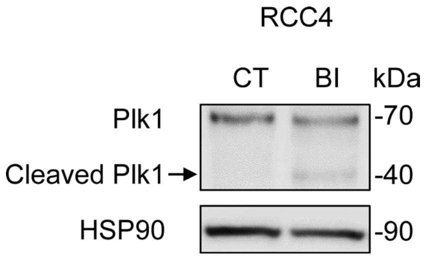

Supplementary Figure 1: BI-2536 induces cleavage of Plk1 in renal cancer cells. RCC4 cells were treated with 25 nM BI-2536 for 48 h. Plk1 cleavage was evaluated by Western blot. Panel is representative of at least 3 independent experiments.

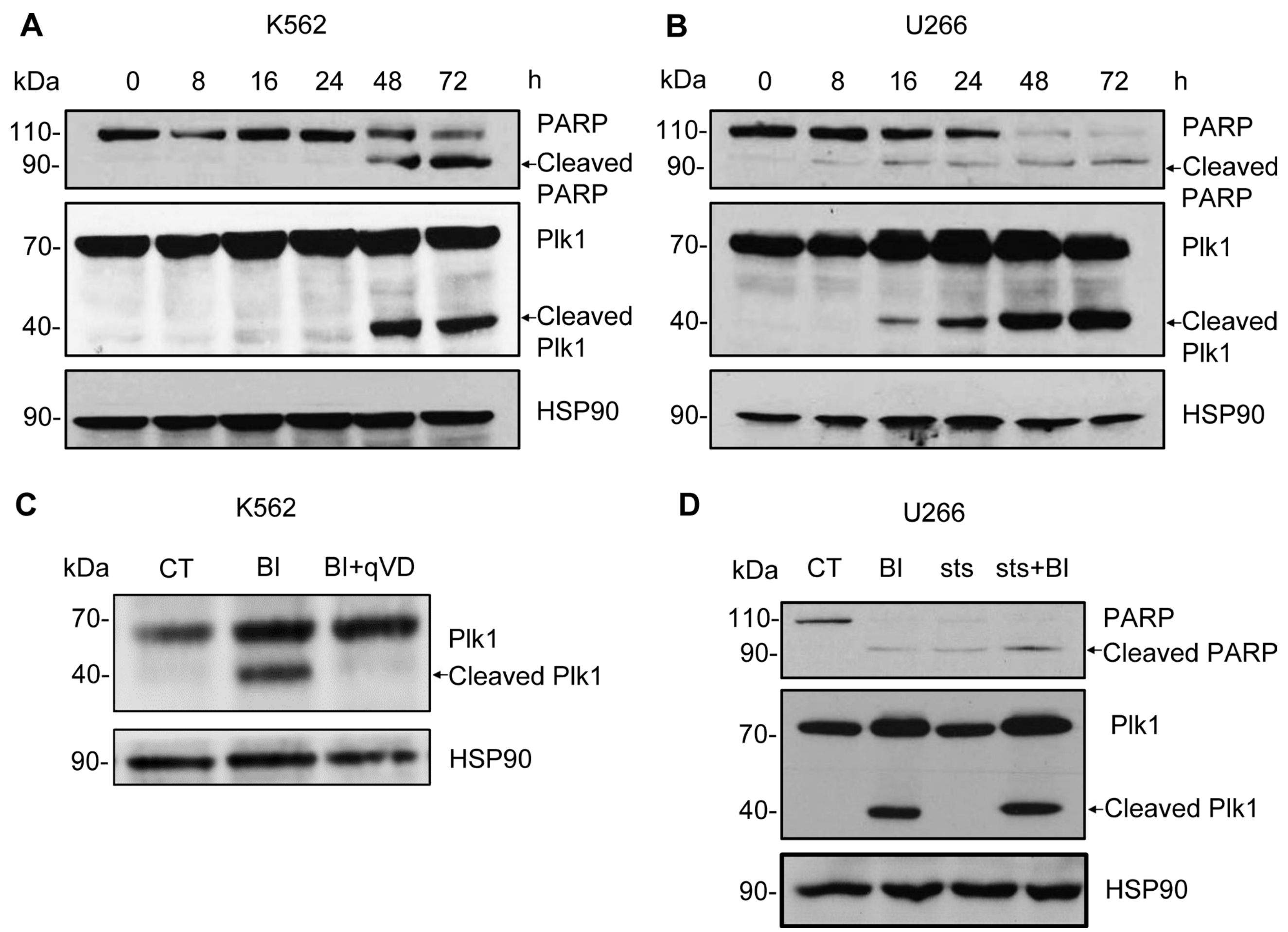

Supplementary Figure 2: BI-2536 induces cleavage of PARP and Plk1. (**A** and **B**) K562 (**A**) and U266 (**B**) cells were treated with 25 nM BI-2536 for different times. PARP and Plk1 cleavage were evaluated by Western blot. (**C**) U266 were treated with 25 nM BI-2536 in presence of 50 μM qVD for 48 h. Plk1 cleavage were evaluated by Western blot. (**D**) U266 were treated with 25 nM BI-2536 with or without 1 μM staurosporin (sts) for 48 h. PARP and Plk1 cleavage were evaluated by Western blot. Panel representative of at least 3 independent experiments.

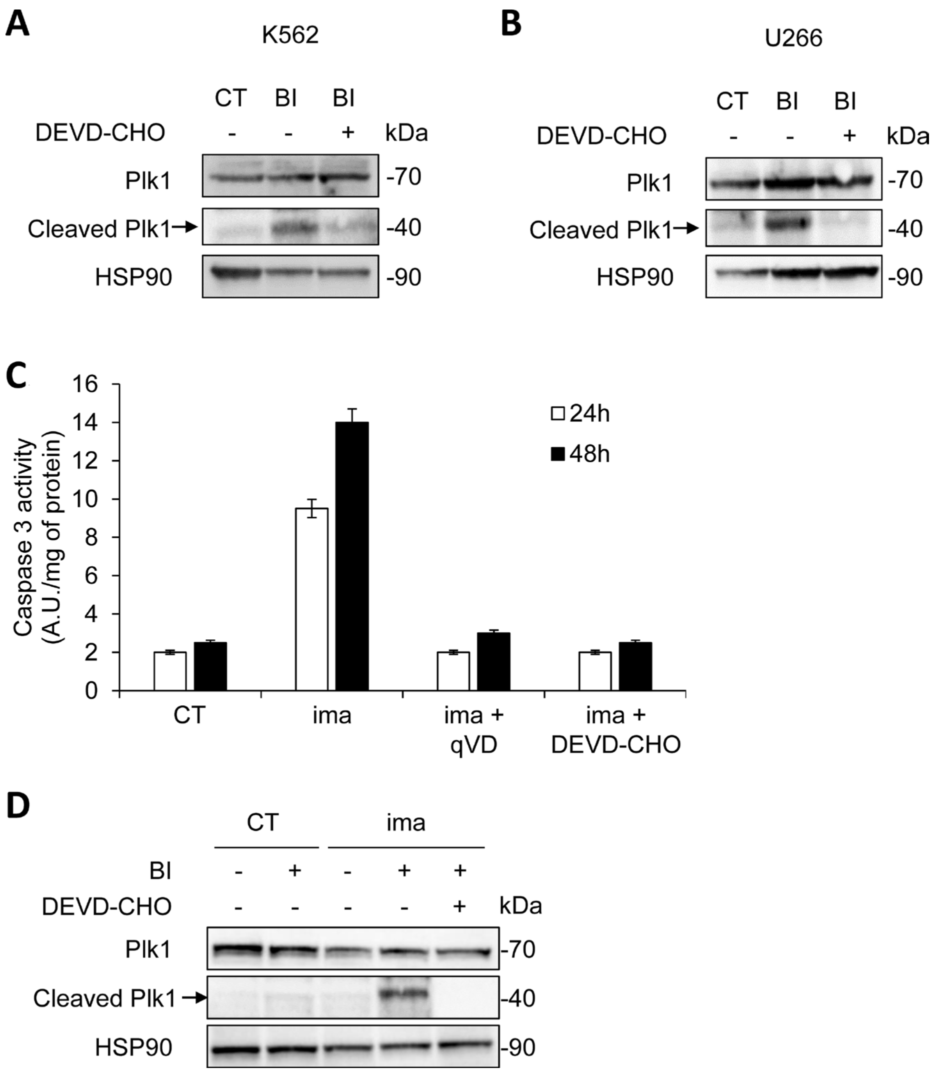

Supplementary Figure 3: BI-2536 induces cleavage of Plk1 by caspase 3. (**A** and **B**) K562 (**A**) and U266 (**B**) cells were treated with 25 nM BI-2536 in the presence of 100 µM DEVD-CHO for 48 h. Plk1 cleavage was evaluated by Western blot. (**C**) K562 were treated with 1 µM imatinib in presence of 50 µM qVD or 100 µM DEVD-CHO for 24 or 48 h. Cells were lysed in caspase buffer and caspase-3 activity was evaluated in quadruplicate using 0.2 mM Ac-DEVD-AMC as substrate. Results are expressed as arbitrary units (A.U.)/min per mg of protein. (**D**) K562 cells were pre-treated with 1 µM Imatinib (ima) in the presence of 100 µM DEVD-CHO for 24 h. Then, cells were treated with 25 nM BI-2536 for 6 h. Plk1 cleavage was evaluated by Western blot. Panel representative of at least 3 independent experiments.

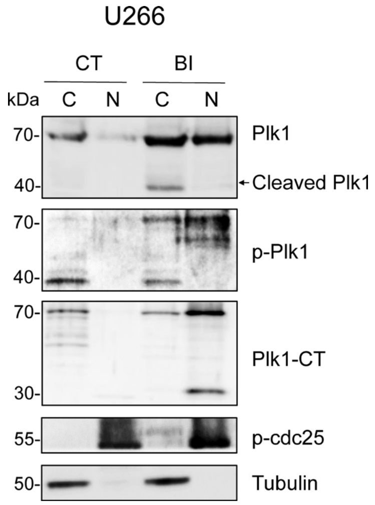

Supplementary Figure 4: Plk1 location in U266 cells. U266 cells were treated with 25 nM BI-2536 for 24 h. Next, cell extracts were separated into cytosol and nuclear-enriched fractions and Plk1 location was assessed by Western blot in the different fractions. Tubulin serves as both loading control and validation of the cytoplasmic fraction.

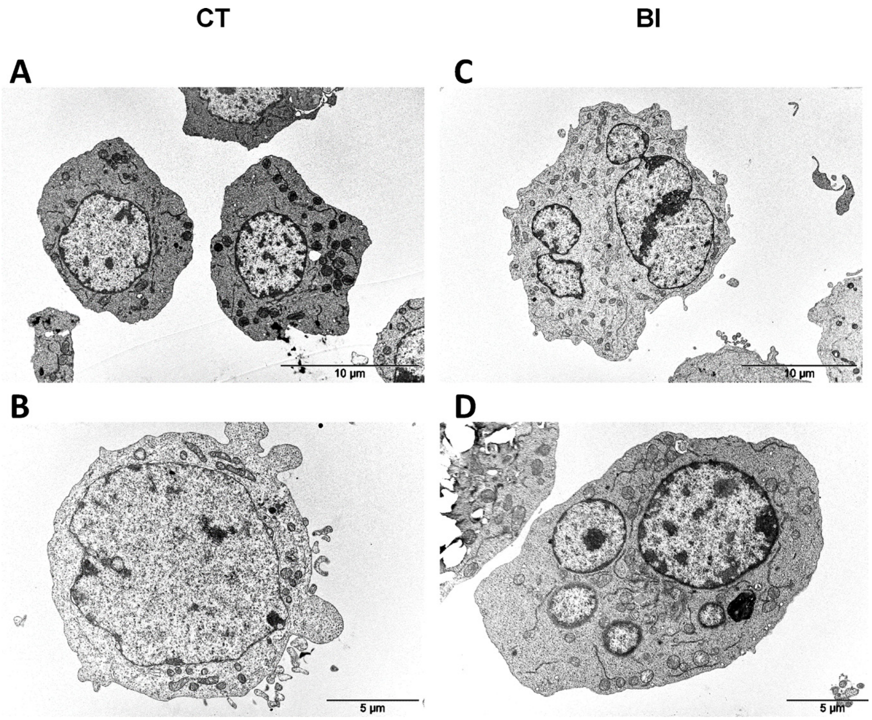

Supplementary Figure 5: BI-2536 induces mitotic catastrophe. (**A–D**), K562 cells were treated with 25 nM BI-2536 for 16 h. Next, cells were fixed and analysed by transmission electron microscopy.

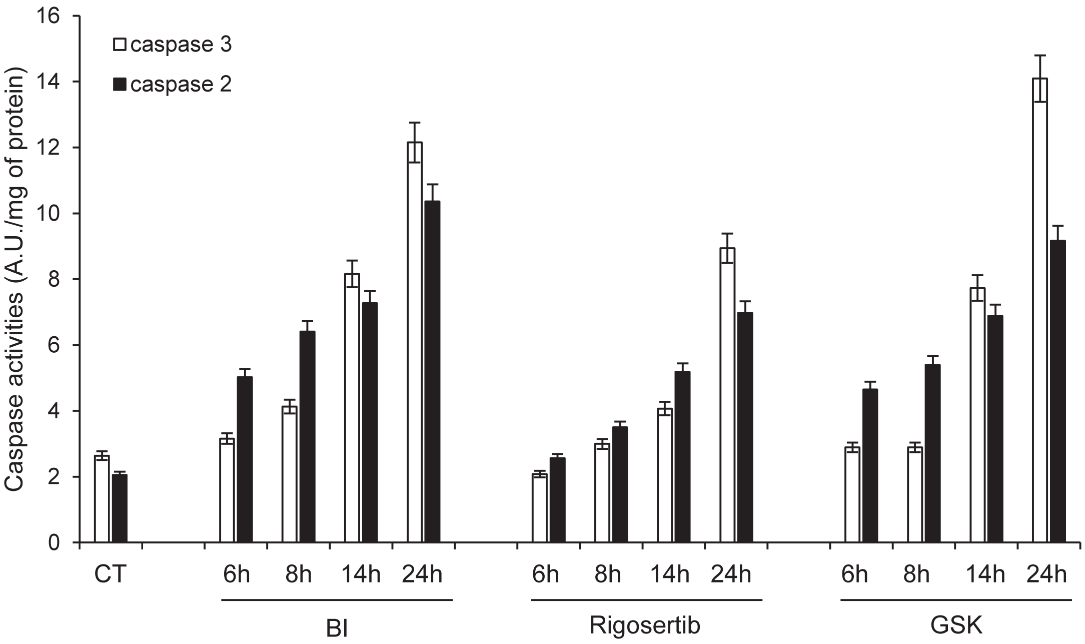

Supplementary Figure 6: BI-2536 activates caspase 2 and 3 in K562 cells. (**A** and **B**) K562 cells were treated with 25 nM BI-2536, GSK-431363, and Rigosertib for 6 to 24 h. Cells were lysed in caspase buffer and caspase-2 and -3 activities were evaluated in quadruplicate using 0.2 mM Ac-VDVAD-AMC or Ac-DEVD-AMC respectively as substrates. Results are expressed as arbitrary units (A.U.)/min per mg of protein.

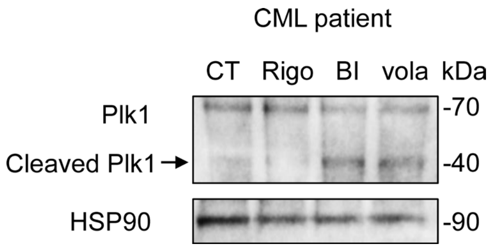

Supplementary Figure 7: BI-2536 and Volasertib induces cleavage of Plk1 in CML primary cells. Primary cells of CML patient were isolated from bone marrow. Next, cells were treated with 250 nM BI-2536, Volasertib or Rigosertib for 48 h. Plk1 cleavage was evaluated by Western blot.
